# Short and Long-Term Outcomes of Transcatheter Aortic Valve Implantation in the Small Aortic Annulus: A Systematic Literature Review

**DOI:** 10.3390/jpm14090937

**Published:** 2024-09-02

**Authors:** Francesco Cabrucci, Massimo Baudo, Yoshiyuki Yamashita, Aleksander Dokollari, Serge Sicouri, Basel Ramlawi

**Affiliations:** 1 Department of Cardiac Surgery Research, Lankenau Institute for Medical Research, Main Line Health, Wynnewood, PA 19096, USA; baudom@mlhs.org (M.B.); yamashitay@mlhs.org (Y.Y.); adokollari@sbgh.mb.ca (A.D.); sicouris@mlhs.org (S.S.); ramlawib@mlhs.org (B.R.); 2 Department of Cardiac Surgery, Lankenau Heart Institute, Main Line Health, Wynnewood, PA 19096, USA; 3 Department of Cardiac Surgery, St. Boniface Hospital, University of Manitoba, Winnipeg, MB R2H 2A6, Canada

**Keywords:** TAVI, small aortic annulus, self-expandable valves, balloon-expandable valves, hemodynamic performance, clinical outcomes

## Abstract

Transcatheter aortic valve implantation has revolutionized the treatment of aortic stenosis. The small aortic annulus is one of the most challenging aspects of aortic stenosis treatment and since the beginning, TAVI has shown promising results in this subgroup of patients. This systematic literature review aims to investigate the short and long-term outcomes of TAVI in the small aortic annulus. The literature was meticulously screened for this topic until April 2024 using the PRISMA guidelines. Technical aspects, characteristics of this subgroup of patients, hemodynamic performances, and outcomes are discussed. The importance of device selection has shown up, with insight into the differences between self-expandable and the balloon-expandable valves. Two special populations were also taken into account: outcomes of TAVI in the small aortic annulus with bicuspid aortic valve and extra-small aortic annulus. The last 10 years have been paramount in technological advancements, bringing TAVI to broader use in this population. While several important trials underscored the usefulness of TAVI in the small aortic annulus population, the clinical practice still lacks consensus on the ideal device, and the outcomes are debated. The pivotal role of TAVI in this context needs to be addressed with a patient-tailored approach to optimize patient care.

## 1. Introduction

As the most common valvular disease treated by interventional cardiologists and cardiac surgeons, there is growing interest today in severe aortic stenosis (AS). Transcatheter aortic valve implantation (TAVI) has revolutionized the treatment of AS. TAVI has become a safe and indispensable treatment option, with recent advances addressing key issues such as vascular complications, pacemaker implantation, paravalvular leakage rates, and, last but not least, it is trying to address the small annular size in AS.

The small aortic annulus (SAA) has always been one of the most challenging aspects of AS treatment: patient–prosthesis mismatch (PPM) is a true nightmare, a subtle failure in surgical aortic valve replacement (SAVR) that surgeons have learned to know over the years. Conversely, it was evident the potential of TAVI in patients with an SAA has been evident since the beginning of the transcatheter heart valve (TVH) era.

In 2012, the first-in-man TAVI of a 20 mm Edwards SAPIEN XT valve performed by Rodés-Cabau et al. paved the way in the challenging field of AS in the small and extra-small aortic annulus [1].

In the last decade, the tremendous advancement in THVs has brought a huge armamentarium to counteract the obstacles encountered in addressing AS in the context of an SAA. Despite these improvements, we are still far from achieving excellent outcomes in this cohort of patients and a lot of gaps need to be addressed in the next few years.

This systematic review aims to provide insight into the field of TAVI in an SAA by looking at the outcomes achieved in the last decade and the innovations in terms of devices and clinical strategies that can lead to better results, and giving a perspective into the future to address the potential gaps in knowledge.

## 2. Definition of the SAA in the Context of TAVI

The definition of the SAA has been widely debated regarding the tool used for sizing (transesophageal echocardiography vs. CT) and the cut-off values. For several years, the cut-off measures defining an SAA have been arbitrary, and the majority of the literature used not only different cut-off values but also relied on different structures [2].

It was first proposed to use a <20 mm aortic annulus to define the SAA population [3]. Then, in 2014, Rodés-Cabau et al. used a cut-off of <23 mm to reach a milestone with the first randomized clinical trial (RCT) facing SAVR and TAVI in an SAA (VIVA trial) [4]. The need for relatively precise cut-off values has emerged in the work by Schmidt et al., in which they divided their real-world large population into quintiles based on the aortic annulus area and observed the outcome trends and the different devices’ performances [5]. Today, the definition of an SAA in the context of TAVI can still vary, but it generally refers to an annular perimeter of less than 72 mm or an area ranging from less than 400 mm^2^ sized on CT, as reported in the TAVI-SMALL registry [6] and TAVI-SMALL 2 registry [7], or less than 430 mm^2^, as considered in the just-published SMART trial [8]. A summary of key studies is provided in Table S1.

## 3. Materials and Methods

This review was carried out following the Preferred Reporting Items for Systematic Reviews and Meta-Analyses (PRISMA) guideline and it was registered on Open Science Framework (OSF) at osf.io/ar854 [9].

The following databases were searched for studies meeting our inclusion criteria and published by 27 April 2024: PubMed/MEDLINE and Google Scholar. We searched for the following terms: [“Transcatheter Aortic Valve Replacement” OR “Transcatheter Aortic Valve Implantation” OR “TAVI” OR “TAVR”] AND [“small aortic annulus” OR “small size aortic annulus”] AND [“outcomes’’ OR “results”]. The following steps were taken for study selection: (1) identification of titles of records through database search; (2) removal of duplicates and articles in non-English; (3) screening and selection of abstracts; (4) assessment for eligibility through full-text papers.

### 3.1. Inclusion Criteria

Studies were included if any of the following criteria were met: (1) reported short or long-term outcomes of TAVI in a patient population with an SAA or a general population but the data were stratified according to aortic annulus size; (2) reported short or long-term hemodynamic performances in TAVI in a patient population with an SAA or a general population but the data were stratified according to aortic annulus size; (3) reported comparison between different types of THV devices in a patient population with an SAA or a general population but the data were stratified according to aortic annulus size; (4) reported comparison in outcomes and/or hemodynamic performances between SAVR and TAVI in a patient population with an SAA or a general population but the data were stratified according to aortic annulus size.

### 3.2. Exclusion Criteria

Studies were excluded if any of the following criteria were met: (1) reported outcomes of TAVI in a general population without stratification according to aortic annulus size; (2) grouped outcomes of TAVI on native valve and Valve-In-Valve; (3) not published in the English language; (4) not published in a peer-reviewed journal; (5) conference abstracts; and (6) case reports.

### 3.3. Data Collection

The data collection was done by 27 April 2024. One author (FC) screened the articles and reviewed them three times. The results were reviewed by other co-authors (MB; YY). Discrepancies were arbitrated by other senior authors to achieve consensus (SS; AD; BR).

**Figure 1:**
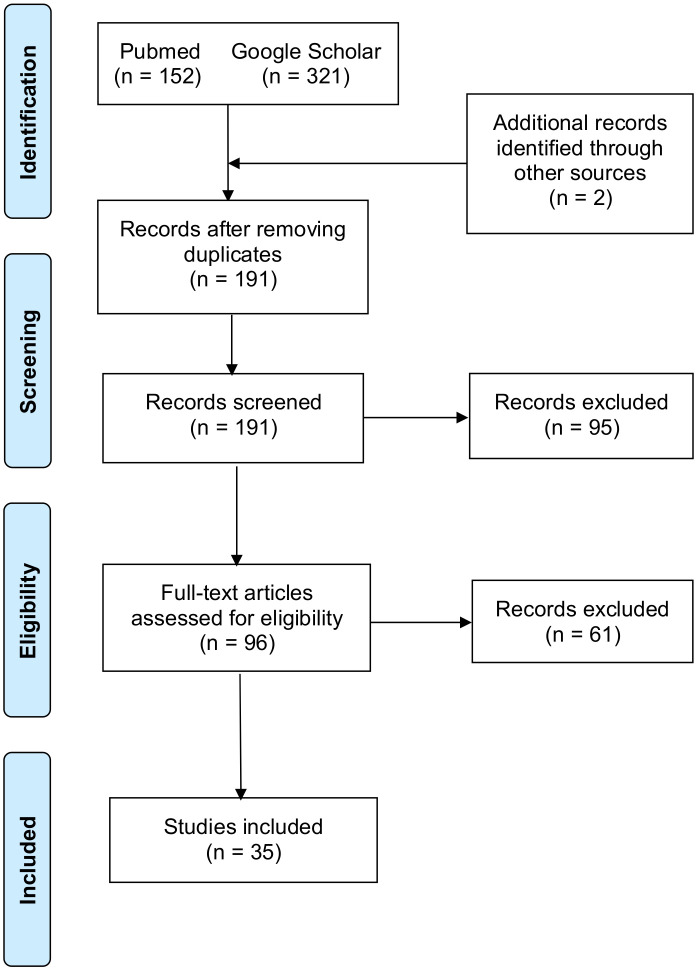
PRISMA 2020 flow diagram of the included studies.

The data collection process is shown in the PRISMA flow diagram for systematic review in Figure 1.

## 4. Technical Aspects and Characteristics of the SAA Population

Performing TAVI in patients with an SAA presents several challenges and considerations. These include the need for patient selection, device implantation optimization, and a deep understanding of the features of this subset of patients [10].

### 4.1. Technical Aspects

The challenges posed by an SAA in the context of AS have been addressed through various procedural techniques. These include various types of aortic root enlargement and several types of valves [11].

SAVR has also seen improvements in valve designs and surgical techniques, although the ideal prosthetic valve for an SAA remains elusive [12]. Stentless bioprostheses have been created to address hostile anatomy and Repossini et al. performed a propensity-matched analysis of 142 patients who underwent SAVR with stentless Freedom SOLO (FS) vs. TAVI in patients with a <23 mm aortic annulus in an intermediate risk cohort. They found a mortality of 2.1% for FS and 6.3% for TAVI (*p* = 0.02), and a moderate to severe paravalvular leak (PVL) of 3.5% for TAVI and 0.7% for FS, but the FS mean gradient was 10.8 ± 5.9 mm Hg vs. 10.7 ± 6.9 mm Hg for TAVI (*p* = 0.88) [13].

Lately, traditional aortic annulus enlargement procedures using a pericardial patch have been proposed to overcome aggressive PPM in SAVR because of low mortality and good outcomes [14]. While these findings seem to be in favor of SAVR, in the real world, stentless bioprostheses implantation and aortic root enlargement are time-consuming procedures mastered only by a minority of surgeons and they need a careful selection of patients.

The RCT by Rodés-Cabau et al. was the first to establish the non-inferiority of using TAVI in an SAA compared with SAVR [4]. This study showed that both therapies were a valid alternative for treating patients with AS and an SAA, and for the first time, it suggested the importance of an individualized treatment selection according to baseline characteristics, additional anatomical risk factors, and patient preference. Despite encouraging results, the need to address the burden of PVL (5.7 vs. 0, *p* < 0.01) and other inherent issues of THVs [4] was evident.

TAVI in an SAA presents specific technical challenges, including annulus rupture, acute coronary obstruction, and vascular complications [10,15].

A previous study showed an increased incidence of annulus rupture in patients with a small body size (BSA < 1.75 m^2^) compared with patients with a larger body size (BSA ≥ 1.75 m^2^) (2.3% vs. 0.5%). The technical difficulty becomes even greater when the measured annulus area is <300 cm^2^ as this may consistently increase the risk of annulus rupture due to relative valve oversizing [16,17].

Although the extra-small aortic annulus appears overwhelming, a subanalysis from the OCEAN-TAVI registry showed favorable short-term outcomes. After propensity score matching, 18 pairs of patients were analyzed after they received a 20 mm Sapien-XT (SXT) in case of an annulus size of <314 mm^2^ or a 23 mm SXT in the second group. Despite the EOA being lower (1.22 ± 0.25 vs. 1.44 ± 0.37 cm^2^, *p* = 0.02), the mean gradient (15.4 ± 4.1 vs. 12.2 ± 4.8 mmHg, *p* = 0.04) and moderate PPM (31.6% vs. 7.9%, *p* < 0.01) were higher in the 20 mm SXT group, and the rate of severe PPM was similar between the two groups (0% vs. 0.4%, *p* = 1.00) [18].

The smaller size of the aortic root complex carries an inherited decrease in height in the coronary take-off from the Valsalva sinuses. Acute coronary obstruction in an SAA after TAVI is a rare but potentially life-threatening complication. It is often caused by the displacement of the calcified native valve leaflets over the main coronary ostia, particularly the left coronary artery ostium [19].

This complication can also occur due to inappropriately high positioning of the sealing cuff or the supporting stent of an implanted valve [20].

As stated above, patients who have an SAA tend to have a smaller anatomy and consequently small femoral arteries. Navigating small femoral arteries is challenging and vascular complications are a serious issue in transfemoral TAVI associated with significantly increased patient morbidity and mortality [10]. Despite all these challenges, TAVI has always shown promise in treating the SAA cohort, with newer-generation, lower profile TAVI systems being particularly beneficial for patients with a smaller anatomy [10]. A summary of key studies is provided in Table S1.

### 4.2. Characteristics of the SAA Population

Patients with an SAA are mostly women who tend to be older and more fragile at the time of severe AS diagnosis [4]. They also have a higher percentage of more challenging features of AS like low-flow, low-gradient AS, and it is well-known that they have had less left ventricular remodeling after TAVI.

Female patients tend to have small body sizes and consequently smaller aortic roots, and smaller femoral arteries. As stated previously, all these characteristics carry an intrinsic risk of annular rupture, an increased probability of coronary obstruction, and vascular complications.

Thus, the above-mentioned features give an estimation of how challenging it can be to treat AS in the setting of an SAA in clinical practice. Interventional cardiologists and surgeons, just after sizing a very small annulus on the CT, would expect an old and fragile lady, with small vascular access, and medium to low coronary take-off, to pose the basis for a challenging case. As much as all this may sound overwhelming, the WIN-TAVI registry demonstrated that women with AS who underwent TAVI with the implantation of a small THV had similar 1-year outcomes as those receiving a non-small THV [21]. In more detail, the occurrence of the Valve Academic Research Consortium-2 (VARC-2) efficacy endpoint was similar between the two groups (16.0% vs. 16.3%, *p* = 0.881), and the group treated with a small TAVI also had a lower rate of PVL.

In addition, the importance of sex in patients with an SAA has also been addressed [22,23]. Medranda et al. confirmed a higher prevalence of females with an SAA undergoing TAVI, which was non-significantly associated with higher mortality (30-day: 2.1% vs. 0.8%, *p* = 0.631; 1-year: 6.3% vs. 1.7%, *p* = 0.118) [22]. After matching 99 pairs of men and women from the TAVI-SMALL 2 registry, it has been demonstrated that there were no differences in pre-discharge severe PPM and mortality (8.5 vs. 10.9%, respectively, *p* = 0.586), but there was a significant association in EOA after TAVI in the two groups (women vs. men 0.86 ± 0.25 1.01 ± 0.26, *p* = 0.006) [23]. A summary of key studies is provided in Table S1.

## 5. Clinical Outcomes and Hemodynamic Performance

Surgical or percutaneous treatment of AS carries always an intrinsic risk of PPM, and this is much more evident in the SAA population. PPM is defined as an indexed effective orifice area < 0.85 cm^2^/m^2^ and the risk factors are a large BSA and SAA [24]. According to VARC-3 criteria, PPM is identified in patients with a body mass index ≥30 kg/m^2^ if iEOA ≤ 0.70 cm^2^/m^2^, and severe PPM if iEOA is ≤0.55 cm^2^/m^2^. For patients with a body mass index <30 kg/m^2^, PPM is considered with iEOA < 0.85 cm^2^, and severe PPM is considered if iEOA is <0.65 cm^2^/m^2^ [25].

Since the first trials, the appeal of TAVI was not only to reduce surgical trauma compared to SAVR but also the possibility of reducing the PPM and achieving a better hemodynamic performance [26].

In the SAVR population, and in the SAA cohort in particular, PPM is a real burden, and it has been demonstrated to impact short and long-term outcomes due to several factors: less regression of left ventricular hypertrophy, less improvement in patient functional status, and a 1.8-fold increase in mortality if severe PPM is present [27,28].

The mechanism of action of TAVI carries intrinsic advantages over SAVR: the lack of the sewing ring and the systematic oversizing to deploy the valve leads to a less crowded aortic root and thus to superior hemodynamic performance [29].

In the context of the SAA, these concepts started to be investigated with the first-generation TAVI devices vs. SAVR approximately 10 years ago and they have been already described above [4,13]. The use of second-generation balloon-expandable valve (BEV) SXT has been retrospectively investigated over SAVR by Kamioka et al. In this paper, over a follow-up of two years, in a cohort of 94 patients (TAVI = 35 and SAVR = 59), TAVI showed a decreased incidence of moderate to severe PPM (2.9% vs. 22.0%, *p* = 0.01) and a comparable rate of mortality (7.1% vs. 15.3%, *p* = 0.81). However, the occurrence of PVL remained an issue in the TAVI group (4.3% vs. 0%, *p* = 0.02) [30].

Another large series of two groups of 352 patients after 1:1 case-matching compared TAVI, either BEVs or self-expandable valves (SEVs), with SAVR in a population with a mean annulus diameter of 19.2 ± 0.3 mm. At 1 month, TAVI outperformed the SAVR group by exhibiting a lower mean gradient (12 ± 7 mm Hg vs. 15 ± 6 mm Hg, *p* < 0.001), larger effective orifice area (EOA) (1.46 ± 0.39 cm^2^ vs. 1.25 ± 0.37 cm^2^, *p* < 0.001), and a lower rate of severe PPM (14% vs. 24%, *p* = 0.001). Also in this case, the TAVI group had more PVL (2.5% vs. 0%) [31].

By this time, the potential of TAVI in the SAA population was clear, yet a lot of gaps in evidence remained to be addressed: the long-term outcomes, the right patient selection, and, above all, the device selection.

### 5.1. The Importance of Device Selection

The world of THVs was changing fast and so was the technology of TAVI devices. Despite several studies failing to show the superiority of SEVs versus BEVs, the comparison between these two groups depicted different advantages in clinical practice [32,33]. While SEVs were showing a greater EOA and better hemodynamic performances, BEVs were exhibiting lower rates of vascular complications and permanent pacemaker implantation (PPI) [32,33].

The first trial comparing SEVs and BEVs in the context of the SAA was done by Lee Y et al. [34]. A cohort of 70 patients (SEV = 45, BEV = 25) with a minimal annulus diameter of < 21mm was observed for 1 year. The SEV group presented a larger EOA (1.75 ± 0.42 vs. 1.46 ± 0.28 cm^2^, *p* = 0.002) and a decreased incidence of ≥moderate PPM (8.9% vs. 36.0%, *p* = 0.009). No differences were evident in the two groups in terms of PVL, vascular complications, and mortality [34].

The multicenter OCEAN-TAVI registry analyzed 576 patients with an SAA undergoing transfemoral TAVI using third-generation SEVs (Evolut R/Pro) and BEVs (Sapien 3) [35]. In this registry, Evolut R/Pro seemed to be superior to Sapien 3 in hemodynamic performance (iEOA 1.20 vs. 1.08 cm^2^/m^2^, *p* < 0.001; PPM 9.2% vs. 20.9%. *p* < 0.001) up to 1 year of follow-up. Nevertheless, all-cause mortality was similar between the two groups (overall 2.9%) [35].

Two RCTs were published in this regard [8,36]. The CHOICE trial, which included older-generation valves, showed that SEVs were associated with lower rates of PPM compared to BEVs [36]. For newer-generation THVs, the SMART trial revealed that SEVs were not inferior to a BEV with respect to clinical outcomes and was superior with respect to bioprosthetic valve dysfunction through 12 months [8]. These two valves have been compared in the context of the SAA by several research groups all over the world, and equivalent outcomes at 1-year follow-up have been reported [37,38,39,40,41,42,43].

### 5.2. An Insight into the SEV World

The TAVI-SMALL registry was the first multicenter trial to investigate the hemodynamic performances of different types of SEV in the subset of SAA patients [6]. This study analyzed the outcomes of different types of supra-annular and intra-annular SEVs, in particular, Evolut R, n = 397; Evolut PRO, n = 84; ACURATE, n = 201; and Portico, n = 177. While gradients were consistently low in every group, a slight advantage was noted in favor of Evolut R, Evolut Pro, and ACURATE prostheses compared to Portico [6]. A statistically significant difference was also found in the rate of more than mild PVL (Evolut PRO 3.6%, Evolut R 11.8%, ACURATE 9%, and Portico 19.2%), while no differences were found in the other variables [6].

Eckel et al. analyzed specifically the procedural short-term outcomes of the ACURATE *neo/neo 2* devices in two German high-volume centers. In this trial, the clinical and hemodynamic outcomes were compared among patients with implantations in adherence to the recommended sizing (on-label *n* = 529) and even below the applicable instructions for use (off-label *n* = 125) [44]. Despite that in the off-label group no device-related deaths occurred, the 30-day all-cause mortality was higher (6.5% vs. 2.3%, *p =* 0.036). Moreover, after multivariate analysis, a greater cover index (Odds ratio OR 3.26), deep implantation (OR 2.25), and severe calcification (OR 2.07) were found to be independent predictors of PPM [44].

The Portico SEV was compared to the SXT by Del Trigo et al. [45]. The authors concluded that TAVI using the Portico system demonstrated comparable short-term hemodynamic outcomes to the SXT system in patients with severe AS and SAA.

### 5.3. Contemporary Long-Term Outcomes in Large Trials

In the last few years, several large studies have been published trying to address the long-term outcomes of TAVI in an SAA [5,7,46,47,48].

In one of the largest multicenter real-world TAVI registries, Schmidt et al. analyzed 2609 patients who were treated with SAPIEN 3 (*n*  =  1146), ACURATE Neo (*n*  =  649), Evolut R (*n*  =  546), or Evolut Pro (*n* =  268) THVs, performing an accurate analysis based on the quintiles of aortic annulus size [5]. This study powerfully stated again that SEVs provided superior hemodynamics in terms of larger EOA, and lower gradients compared to BEVs, in particular in the SAA subgroup. Severe PPM was less frequent in SEVs, and, interestingly in this case, the rate of PVL ≥ moderate was comparable for both types of devices in an SAA [5].

Kornyeva et al. investigated the 3-year outcomes of a group of 507 patients who underwent TAVI with an SAA [48]. After the propensity match score, they obtained 192 pairs treated either with SEVs or BEVs. The VARC -3 device success was 86% in SEVs and 84% in BEVs (*p* = 0.8). The rate of moderate (31% vs. 20%, *p* < 0.001) and severe PPM (18% vs. 9%, *p* < 0.001) was higher in BEVs compared to SEVs.

Despite that BEV implantation in patients with an SAA was associated with a two-fold higher incidence of pre-discharge severe PPM compared to SEV implantation, survival at 3 years was similar in the two groups (72%, *p* = 0.9). However, patients with an absence of pre-discharge PPM had a higher 3-year survival compared to patients with ≥moderate PPM (77% vs. 67%, *p* = 0.03) [48].

These findings have been further confirmed at 5 years by Okuno et al. [47]. While the rate of moderate to severe PPM was consistently higher in the BEV group compared to the SEV group at discharge (51.8% vs. 19.7%; *p* < 0.001), the hemodynamic advantage of self-expanding THVs was not associated with better clinical outcomes compared with balloon-expandable THVs up to 5 years. Of note, disabling strokes occurred more frequently in patients with a self-expanding THV than those with a balloon-expandable THV (6.6% vs. 0.6%; *p* = 0.030) [47]. A summary of key studies is provided in Table S1.

## 6. Outcomes in “Special” Populations

### 6.1. TAVI in SAA in the Bicuspid Aortic Valve Population

TAVI is feasible in patients with a bicuspid aortic valve (BAV), but whether annular size may influence the outcomes in this subset of patients remains unknown [49].

The BEAT registry is a multicenter database of consecutive BAV AS undergoing TAVI which divided patients according to annular dimension: small annulus 15.5% (area < 400 mm^2^ or perimeter < 72 mm), medium annulus 45.3% (area ≥ 400 and < 575 mm^2^, perimeter ≥ 72 mm and < 85 mm), or large annulus 39.3% (area ≥ 575 mm^2^ or perimeter ≥ 85 mm). The primary endpoint was VARC-2 device success; the potential interactions between annulus size and the different THV devices were also investigated [50]. While the large annular size group had an expected larger aortic valve area and a lower mean gradient, no differences in clinical outcomes were observed according to annular size or THV type; thus, TAVI in BAV patients was feasible irrespective of the annular size [50].

### 6.2. The “Extra-Small” Aortic Annulus

As mentioned above, the favorable short-term outcomes in the extra-small aortic annulus population undergoing TAVI came from the subanalysis of the OCEAN-TAVI registry [18].

A recently published study, based on a multicenter database, analyzed the two-year outcomes in 150 patients with extra-SAA (defined as an aortic annulus area < 280 mm^2^ or perimeter < 60 mm) undergoing TAVI, of which 92.7% were women, and 73.3% received an SEV [51].

At short-term follow-up, VARC-3 criteria were used, and a comparison was also done between SEV and BEV devices. Intraprocedural technical success was 91.3%, with a higher rate in patients receiving a self-expandable THV (96.4% vs. 77.5% with BEV; *p* = 0.001). Overall, 30-day device success was 81.3%, (85.5% with SEV vs. 70.0% with BEV; *p* = 0.032). Severe PPM occurred in 12% overall (9.0% with SEV and 24.0% with BEV; *p* = 0.039), with no impact on all-cause mortality, cardiovascular mortality, or heart failure readmission at the 2-year follow-up [51]. A summary of key studies is provided in Table S1.

## 7. Future Directions

TAVI has reached several milestones in the last decade and, with regard to the small aortic annulus, has unleashed an extreme potential in terms of safety, hemodynamic performances, and short and long-term outcomes. Although the results obtained are exciting, the road to achieving excellent results is still long ahead.

On one side, while the technological innovations in THVs are huge, and new TAVI devices are released for clinical use quite often, there is an urgent need to understand and evaluate those devices in the clinical field. The most recent large trials in the SAA group investigated not only the differences between self-expandable and balloon-expandable valves but also the outcomes related to the intra-annular and supra-annular positioning and the role of post-dilatation [7,52].

On the other side, several gaps in knowledge need to be filled. One recent report questioned again whether SAVR in the SAA population provides a better LV mass regression than TAVI with a comparable rate of PPM [53]. The early results of the ongoing VIVA TRIAL showed no differences between TAVI and SAVR in small aortic annuli [4]. Finally, yet importantly, the durability of the new devices needs to be carefully evaluated in the long term. Despite older devices having shown excellent durability in terms of structural valve dysfunction and bioprosthetic valve failure in the general population, the SAA subgroup needs insight analysis due to the intrinsic challenges in the case of reintervention [54].

## 8. Conclusions

TAVI has proven to be an excellent tool in treating AS in the context of the SAA. The last 10 years have been paramount in technological advancements, bringing TAVI to a broader use. Several important trials underscored the usefulness of TAVI in small aortic annuli and investigated the importance of device selection. Despite SEVs and supra-annular valves having been associated with lower rates of PPM and good hemodynamic results, the importance of careful patient selection and the valve choice, based on accurate sizing of the aortic annulus, remain the best predictors for a favorable outcome. Therefore, the pivotal role of TAVI in the context of small aortic annuli needs to be addressed with a patient-tailored approach to optimize patient care.

## Data Availability

Data supporting reported results can be found in PubMed/MEDLINE and Google Scholar.

## Supplementary Materials

The following supporting information can be downloaded at: https://www.mdpi.com/article/10.3390/jpm14090937/s1, Table S1: Authors, Inclusion criteria, hemodynamic performances, and conclusion of the analyzed studies in chronological order.
